# Role of NRF2 in Pathogenesis of Alzheimer’s Disease

**DOI:** 10.3390/antiox13121529

**Published:** 2024-12-13

**Authors:** Ching-Tung Chu, Akira Uruno, Fumiki Katsuoka, Masayuki Yamamoto

**Affiliations:** 1 Department of Biochemistry and Molecular Biology, Tohoku Medical Megabank Organization, Tohoku University, Sendai 980-8573, Japan; chu.chingtung.t3@dc.tohoku.ac.jp (C.-T.C.); akira.uruno.a7@tohoku.ac.jp (A.U.); 2 Department of Integrative Genomics, Tohoku Medical Megabank Organization, Tohoku University, Sendai 980-8573, Japan; kfumiki@tohoku.ac.jp; 3 Advanced Research Center for Innovations in Next-Generation Medicine, Tohoku University, Sendai 980-8573, Japan

**Keywords:** Alzheimer’s disease, oxidative stress, microglia, inflammation, NRF2, KEAP1

## Abstract

Alzheimer’s disease (AD) is a polygenic, multifactorial neurodegenerative disorder and remains the most prevalent form of dementia, globally. Despite decades of research efforts, there is still no effective cure for this debilitating condition. AD research has increasingly focused on transcription factor NRF2 (nuclear factor erythroid 2-related factor 2) as a potential therapeutic target. NRF2 plays a crucial role in protecting cells and tissues from environmental stressors, such as electrophiles and reactive oxygen species. Recently, an increasing number of studies have demonstrated that NRF2 is a key regulator in AD pathology. NRF2 is highly expressed in microglia, resident macrophages in the central nervous system, and contributes to neuroinflammation, phagocytosis and neurodegeneration in AD. NRF2 has been reported to modulate microglia-induced inflammation and facilitate the transition from homeostatic microglia to a disease-associated microglia subset. Genetic and pharmacological activation of NRF2 has been demonstrated to improve cognitive function. Here, we review the current understanding of the involvement of NRF2 in AD and the critical role that NRF2 plays in microglia in the context of AD. Our aim is to highlight the potential of targeting NRF2 in the microglia as a promising therapeutic strategy for mitigating the progression of AD.

## 1. Introduction

Alzheimer’s disease (AD) is a neurodegenerative disorder characterized by the deposition of amyloid β (Aβ) fibrils in the brain, leading to the formation of plaques and TAU-associated neurofibrillary tangles (NFTs), which ultimately result in the degeneration of the brain cells. These pathological features are the primary causes of dementia [1]. More than 55 million people suffer from dementia worldwide, with nearly 10 million new cases reported each year. AD is the most prevalent form of dementia, accounting for 60–70% of cases. In 2019, global spending on dementia amounted to 1.3 trillion US dollars, with 50% of these costs attributed to caregiving. On average, caregivers provide five hours of daily support and supervision [2]. Therefore, it is crucial to delay or slow the onset and progression of AD. It is also important to alleviate its debilitating symptoms to eventually eradicate the disease and prevent an escalating public health crisis.

A key target in combating AD is the KEAP1-NRF2 system. NRF2 (nuclear factor erythroid 2-related factor 2) is a transcription factor which is essential for protecting cells against environmental stressors, such as electrophiles and reactive oxygen species (ROS). NRF2 is primarily regulated by KEAP1 (Kelch-like ECH-associated protein) [2]. Under unstressed conditions, KEAP1 suppresses the transcriptional activity of NRF2 by promoting its constant degradation [2,3]. However, when cells encounter oxidative and/or electrophilic stresses, specific cysteine residues in KEAP1 undergo modifications. As a result, KEAP1-mediated ubiquitination of NRF2 is inhibited, leading to NRF2 stabilization and translocation into the nucleus, where it induces the transcription of a specific set of cytoprotective genes. This regulatory mechanism, known as the KEAP1-NRF2 system, is crucial for protecting cells from environmental stressors [4,5].

A growing body of evidence strongly supports the notion that activating NRF2 signaling reduces oxidative stress and inflammation and protects tissues from oxidative damage [6]. Since oxidative stress, inflammation, and impaired proteostasis are significantly involved in the pathogenesis of AD [7], the KEAP1-NRF2 system has emerged as a promising therapeutic target for the disease [8].

Among the main cell types in the central nervous system (CNS), NRF2 expression levels are highest in microglia, followed by astrocytes, with significantly lower levels in neurons [9,10]. This distribution suggests that the contributions of the NRF2 signaling pathway are cell-type-dependent, with NRF2 likely playing a particularly prominent role in microglia. Microglia are the immune cells in the brain, responsible for maintaining balance in the CNS [11]. These highly heterogeneous cells interact with neurons, astrocytes, and oligodendrocytes [12]. In AD, microglia often cluster and become activated around amyloid plaques [13]. Additionally, microglia play a role in brain development and adjusting synapses, which is vital for learning and memory, linking them closely to AD.

In this review, we aim to provide a comprehensive understanding of the pathology of AD and the molecular basis of the KEAP1-NRF2 system’s contribution to the prevention of AD pathogenesis. We highlight the roles that NRF2 plays in AD, with a specific focus on its activity in microglia and other CNS cells.

## 2. Physiopathology of AD

The hallmark features of AD include extracellular Aβ plaques and intracellular NFTs [14,15]. Aβ, a small peptide, is derived from the abnormal processing of amyloid-β precursor protein (APP). Under normal conditions, APP is cleaved within the Aβ sequence by metalloproteases, preventing Aβ formation [16]. However, in AD, APP is initially cleaved by BACE1 at the beginning of the Aβ sequence. This cleavage produces a C-terminal fragment called C99, which is subsequently cleaved by the γ-secretase complex to generate Aβ [16]. Presenilin-1 (PS1) serves as the main catalytic component of the γ-secretase complex. After formation, Aβ peptides self-aggregate into toxic oligomers and eventually form extracellular plaques. Neurofibrillary tangles consist of hyperphosphorylated TAU, a protein that normally binds to microtubules to stabilize their structure. In AD, TAU becomes hyperphosphorylated, losing its ability to effectively bind microtubules, leading to neuronal dysfunction [17].

Mitochondrial dysfunction is another critical process in AD. Changes in mitochondrial morphology, reduced cytochrome oxidase activity, deficits in metabolic proteins, and disruptions in mitochondrial membrane potential are commonly observed [18,19]. Neurons, which heavily rely on mitochondria for their high metabolic demands, particularly at synapses, become vulnerable to oxidative stress caused by excessive ROS and inadequate antioxidant defenses. This oxidative stress exacerbates neuronal damage and contributes to cognitive decline [18].

Inflammation plays a central role in AD pathology. Chronic inflammation has been linked to an increased risk of dementia, with higher inflammatory marker levels correlating with faster cognitive decline. Animal studies have shown that inflammation induces cognitive impairment, neuronal damage, and synaptic loss [20,21,22]. While acute microglial activation may initially protect neurons, prolonged activation can result in neurotoxicity and enhanced Aβ accumulation [23]. Microglia are drawn to Aβ plaques, where they exhibit increased phagocytic activity. However, chronic activation creates a detrimental feedback loop that exacerbates neurodegeneration [24]. TAU pathology also interacts with inflammation, as microglia may initially help clear TAU aggregates but lose this function over time [25,26]. Additionally, ROS levels can increase inflammatory markers, while inflammation, in turn, amplifies oxidative stress, highlighting the complex interplay between Aβ, TAU, oxidative stress, and inflammation [27].

The role of the brain vasculature in AD has also gained attention [28]. Pericytes, which maintain the extracellular matrix and regulate blood flow, show selective vulnerability in AD patients. Multiple genome-wide association studies (GWASs) have revealed that many AD risk genes are expressed in brain vascular cells [29]. Vascular contributions to AD pathology include impaired neurovascular coupling, blood–brain barrier (BBB) disruption, and reduced clearance of waste products like Aβ. Neurovascular coupling, which adjusts cerebral blood flow to match neuronal metabolic demands, is often disrupted in AD, impairing neuronal function and increasing brain damage [30]. The BBB, a key component of the neurovascular unit (NVU), maintains brain homeostasis by blocking harmful substances and clearing waste metabolites. Its breakdown contributes to cerebrovascular dysfunction and neurodegeneration in AD [31].

## 3. Aspects of NRF2 Biological Activity

### 3.1. Structure and Regulatory Mechanisms of NRF2 Protein

**Figure 1:**
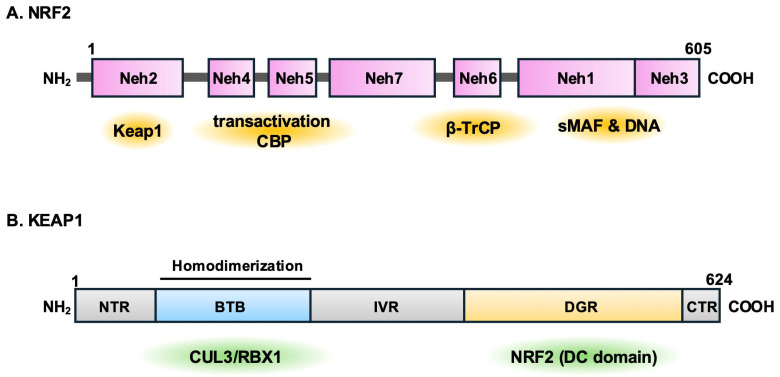
Domain architectures of NRF2 and KEAP1. (**A**). NRF2 (nuclear actor erythroid 2-related factor 2) consists of seven domains, referred to as Neh1 to Neh7, each identified based on their specific biological functions and their homology to other protein domains. Neh stands for Nrf2-Ech homology domain. These domains play the crucial regulatory roles of NRF2 in cellular defense mechanisms, including oxidative stress response and detoxification processes, and anti-inflammation. (**B**). KEAP1 (Kelch-like ECH-associated protein) is composed of five domains. BTB domain and DGR domain are protein–protein interaction domains, separated by an intervening region (IVR). The BTB domain regulates homodimerization of KEAP1 and binding to CUL3 (Cullin3), while the DGR domain and the C-terminal region facilitate the binding with Neh2 domain of NRF2.

NRF2 is a member of the cap’n’collar (CNC) family of transcription factors (Figure 1A), which also includes NRF1, NRF3, and p45 NF-E2. The CNC factors belong to a basic leucine zipper (bZIP) protein family and heterodimerizes with small MAF (sMAF) proteins before binding to specific DNA sequences, such as the antioxidant response element (ARE) or the electrophile response element (EpRE), which are essentially the same sequence and are collectively termed CNC-sMaf-binding element (CsMBE) [5,32,33]. NRF2 is not uniformly but widely expressed throughout the body and is recognized as the key regulator of the cellular antioxidant defense mechanism. Among the CNC family members, NRF2 is considered the primary regulator of detoxification enzyme genes, particularly in metabolic organs and tissues, where its expression is highly induced in response to various stressors [5,32,33]. It controls the expression of numerous antioxidant and detoxification enzymes [5,34], as well as factors involved in maintaining proteostasis [35]. Overall, NRF2 is crucial for protecting cells, tissues, and the body against environmental stressors, including electrophiles and ROS.

To understand how different stressors activate NRF2, a series of structure–function analyses on the NRF2 protein were conducted [32]. NRF2 contains seven functional domains, referred to as NRF2-ECH homology (Neh 1-7) domains, which interact with various factors or cis-acting DNA motifs. (Figure 1A) [2]. The Neh2 domain acts as a degron for NRF2 and is crucial for regulating cellular NRF2 abundance and activity, which is controlled by KEAP1, as will be discussed below. The Neh1 domain is a basic-region leucine zipper domain that facilitates dimerization with the small Maf protein for DNA binding, a structure recently elucidated [36]. The Neh6 domain plays a significant role in interacting with β-TrCP (β-transducin repeat-containing protein) and acts as an auxiliary degron independent of KEAP1. The Neh4 and Neh5 domains are transactivation domains that interact with transcriptional coactivators, including CREB-binding protein (CBP) [37,38,39].

NRF2 is primarily regulated by KEAP1 (Figure 1B) [2]. KEAP1 is a component of the Cullin3 (CUL3)-based ubiquitin E3 ligase and functions as an adapter that facilitates the ubiquitination and subsequent degradation of NRF2. KEAP1 contains three major domains and forms a homodimer through its BTB domain, located at the N-terminal part of KEAP1. The C-terminal region of KEAP1 forms the DC domain, which binds to NRF2. The BTB domain interacts with an E3 ubiquitin ligase complex (RBX1) via the adaptor protein CUL3 (Figure 1B) [3]. This E3 complex facilitates the ubiquitination and constitutive degradation of NRF2 through the 26S proteasome [40].

Under normal conditions, NRF2 is bound to KEAP1 in the cytoplasm through its Neh2 domain, which promotes continuous degradation and suppresses its transcriptional activity [2,3]. However, when cells are exposed to oxidative or electrophilic stress, certain cysteine residues in KEAP1 are modified by ROS or electrophilic compounds. These modifications prevent KEAP1 from ubiquitinating NRF2, allowing newly synthesized NRF2 to translocate into the nucleus and activate the expression of various cytoprotective genes [5].

NRF2 stability in nuclei is also regulated by other mechanisms, such as GSK3β (glycogen synthase kinase 3β)-mediated degradation. GSK3β plays a key role in regulating NRF2 by promoting its degradation. Specifically, GSK3β phosphorylates NRF2 at serine residues within the DSGIS motif in the Neh6 domain [37]. This phosphorylation creates a phosphor-degron that is recognized by the E3 ubiquitin ligase adapter β-TrCP, leading to NRF2 ubiquitination and subsequent proteasomal degradation [37].

### 3.2. Roles of NRF2 in Cytoprotection from Oxidative Stress and Inflammation

**Figure 2:**
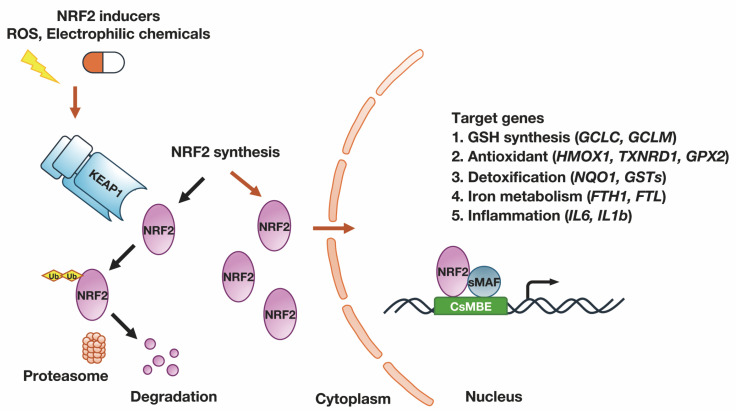
Schematic presentation of the KEAP1-NRF2 system. Under normal conditions (black arrows), NRF2 is bound to KEAP1 in the cytoplasm. KEAP1 targets NRF2 for ubiquitination, leading to its degradation via the proteasomal pathway. Upon exposure to oxidative stress or NRF2 inducers (orange arrows), the KEAP1 undergoes modifications that reduce its ubiquitin ligase activity, resulting in weakening of KEAP1 for ubiquitinating NRF2. Subsequently, newly synthesized NRF2 translocates to the nucleus. In the nucleus, NRF2 binds to CNC-sMaf-binding elements (CsMBEs) or antioxidant response elements (AREs) in the promoter/enhancer regions of target genes. This binding activates the transcription of genes involved in the synthesis of reduced glutathione (GSH), antioxidant defense, detoxification processes, iron metabolism and inflammation.

As summarized in Figure 2, NRF2 upregulates the expression of numerous genes that contain CsMBEs in their regulatory regions [41]. In the early stages of research, NRF2 was found to stimulate the expression of genes involved in antioxidant functions. For instance, NRF2 regulates genes involved in heme metabolism and iron metabolism, such as *HMOX1* (heme oxygenase-1 (HO-1)), *FTH1* (ferritin heavy chain), and *FTL* (ferritin light chain) genes. NRF2 also regulates antioxidant genes, such as *TXNRD1* (thioredoxin reductase 1) and *PRDX1* (peroxiredoxin 1) [42,43,44,45,46].

NRF2 is especially crucial for regulating the production and utilization of a major antioxidant glutathione (GSH). GSH, composed of glutamate, cysteine, and glycine, protects against oxidative stress by detoxifying hydrogen peroxide and organic hydroperoxides via glutathione peroxidase activity [47]. NRF2 activates the basal and inducible expression of enzymes involved in GSH synthesis, such as *GCLC* (glutamate cysteine ligase catalytic subunit) and *GCLM* (glutamate cysteine ligase modifier subunit), glutathione reductase, and various *GSTs* (glutathione S-transferases) [48,49]. It also regulates the cysteine–glutamate exchange transporter *SCL7A11* (solute carrier family 7 member 11), ensuring an adequate supply of cysteine for GSH synthesis [50]. Through these pathways, NRF2 enhances cellular antioxidant capacity.

In addition to antioxidant defense, NRF2 is pivotal in regulating the metabolism and detoxification of xenobiotics and other toxic chemicals that enter the body. Xenobiotics or toxic chemicals undergo three phases of detoxification when they enter the body. In Phase I detoxification reactions, cytochrome P450 enzymes metabolically activate these xenobiotics. The metabolites produced in Phase I often acquire an electrophilic nature, making them suitable substrates for Phase II enzymes These Phase II enzymes, primarily transferase, conjugate hydrophilic moieties—such as GSH, sulfate or glucuronate—to the electrophilic products of Phase I. NRF2 positively regulates the expression of phase II detoxification enzymes, including *NQO1* (NAD(P)H quinone oxidoreductase 1), *GSTs*, and *UGTs* (UDP glucuronosyltransferases) [51,52].

It has been particularly noted that NRF2 downregulates the expression of pro-inflammatory cytokines and chemokines, thereby reducing pathological inflammation [53]. For instance, CsMBEs were found to regulate the expression of inflammatory cytokine genes such as *IL6* and *IL1B*, which encode interleukin 6 (IL-6) and interleukin 1β (IL-1β), respectively, as well as the expression of chemokine genes like *CCL2*, which encodes monocyte chemoattractant protein 1 (MCP-1) [53]. NRF2 suppresses the expression of these proinflammatory cytokine/chemokine genes, dampening the lipopolysaccharide (LPS)-induced expression of *IL6*, *IL1B*, and *CCL2*, thereby reducing pathological inflammation. NRF2 also mitigates inflammation in mouse models of autoimmune diseases, such as Scurfy, NOD mice, and experimental autoimmune encephalomyelitis [54,55,56].

Understanding the interaction between NRF2 and NF-κB (nuclear factor-kB) is critical, as therapeutic strategies targeting this interplay could effectively modulate both oxidative stress and inflammation. The activation of NRF2 can inhibit the NF-κB pathway, thereby influencing cellular responses to oxidative stress and inflammation [57]. NF-κB, including the p65 subunit, also influences cellular responses to stressors and inflammation. The presence of putative NF-κB binding sites in the regulatory region of the *NRF2* gene suggests potential crosstalk between these two regulators in inflammatory processes [57]. Current evidence suggests that NF-κB hinders NRF2 activation through multiple mechanisms: it competes for the co-activator CBP during transactivation, recruits HDAC3 for histone hypoacetylation, and even impedes the DNA binding ability of NRF2. Moreover, NF-κB can promote NRF2 ubiquitination and facilitate KEAP1-mediated degradation, which limit NRF2′s function [58]. This complex interplay between NF-κB and NRF2 is central to the regulation of oxidative stress response and inflammation pathways [59], as these two pathways often converge to regulate cellular responses in various diseases.

Besides the above-mentioned NRF2 target genes, advances in NRF2 research have uncovered new NRF2 target genes, which play very important roles in cytoprotection [44,53,60,61,62,63,64]. Chromatin immunoprecipitation followed by deep-sequencing (ChIP-seq) strongly validated the identification of these newly uncovered NRF2 target genes [63], such as genes involved in the pentose phosphate pathway, glycogen metabolism [64], gluconeogenesis, and autophagy [44,62]. These findings underscore the critical roles of NRF2 also in metabolic regulation and proteostasis [60,61].

### 3.3. Roles of NRF2 in the Homeostatic CNS

NRF2 exerts significant neuroprotective effects in both healthy aging and in response to brain diseases and injuries. In healthy aging, NRF2 activation supports cellular maintenance and resilience by combating oxidative stress, promoting detoxification, and maintaining proteostasis [65]. However, in disease states, such as neurodegenerative disorders, NRF2′s protective role becomes even more critical by mitigating inflammation, oxidative damage, and neurotoxicity [66,67,68].

**Figure 3:**
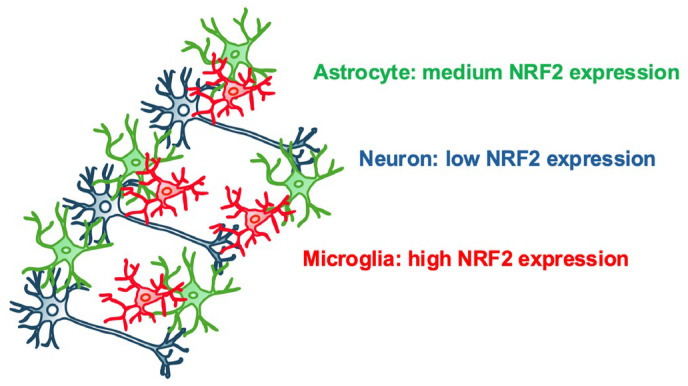
Expression levels of NRF2 in three major central nervous system (CNS) cell types. Microglia show high NRF2 expression, astrocytes exhibit medium NRF2 expression, and neurons display low NRF2 expression.

Immunohistochemical studies have identified that NRF2 is predominantly expressed in glial cells in the brain [69,70,71]. RNA sequencing analyses have further revealed that microglia and astrocytes express high and moderate levels of *NRF2* mRNA, respectively, compared to neuronal cells [9,10,72] (Figure 3). These observations suggest that neurons have a weaker stress response capacity than glial cells. It has been suggested that this expression profile results from the epigenetic inactivation of the *NRF2* gene regulatory region early in cortical neuronal development [73]. Keap1 depletion in astrocytes significantly enhances the expression of *NQO1*, an NRF2 target gene, whereas Keap1 depletion does not enhance it to the same extent in neurons [74]. Similarly, Keap1 depletion induces NQO1 expression in microglia in AD mouse models [75].

Genetic studies have clearly highlighted the pivotal role of NRF2 in maintaining homeostasis, and its loss increases susceptibility to inflammatory, cytotoxic, genotoxic, and neurotoxic insults caused by both endogenous and exogenous stressors [68,76,77,78]. Indeed, mice with genetic deletion of *Nrf2* tend to exhibit vacuolar leukoencephalopathy with widespread astrogliosis compared to age-matched control [76]. *Nrf2*-knockout mice show more severe dopaminergic dysfunction in the basal ganglia and exhibit increased astrogliosis and microgliosis compared to wild-type mice [78]. The loss of NRF2 also increases susceptibility to the neurotoxin 6-hydroxydopamine (6-OHDA) in both cultured neurons and in *Nrf2*-deficient mice [68]. Furthermore, in models of acute brain injury caused by stroke and traumatic brain injury, *Nrf2*-deficient mice experience exacerbated injuries and heightened immune responses [77].

These findings collectively suggest that the KEAP1-NRF2 system plays a crucial role in brain protection and contributes to the maintenance of CNS homeostasis by regulating the expression of cytoprotective enzymes, repressing inflammation and modulating metabolic pathways. Among the various functions of NRF2, its role in modulating microglial dynamics and regulating microglial function seems particularly important.

## 4. The Role of NRF2 in AD

### 4.1. NRF2 Expression Patterns and Its Role in AD Pathogenesis

The accumulation of plaques and tangles triggers local inflammatory responses and oxidative stress, both of which contribute to neurotoxicity. Postmortem examinations of human AD brains reveal significant local inflammation, particularly surrounding extracellular Aβ plaques. The buildup of Aβ and TAU enhances neuroinflammation by activating microglia and astrocytes in AD [79]. Given the antioxidant and anti-inflammatory properties of NRF2, it is reasonable to propose that enhancing NRF2 signaling could reduce the severity of AD pathology.

In the neural cells of a normal hippocampus, NRF2 is primarily detected in the nucleus, indicating its activation. The expression levels of *Nrf2* mRNA in the cortex of wild-type mice exhibit an age-dependent decline [80]. In contrast, in the hippocampal cells of AD patients, NRF2 is predominantly detected in the cytoplasm [81]. Additionally, the expression of NRF2 fluctuates more in the nuclear fraction of hippocampal neurons of AD patients compared to age-matched healthy controls [81]. While NRF2 expression is elevated in the temporal cortices of AD patients, the activities of antioxidant enzymes, such as glutathione peroxidase and catalase—products of NRF2-target genes—are reduced in the superior temporal gyrus [82,83]. A case-control study investigating genetic variations in *NRF2* and *KEAP1* in AD patients found that one haplotype allele of *NRF2* is associated with an earlier onset of AD, suggesting that common variants of the *NRF2* gene may influence disease progression [84]. These observations suggest a strong link between NRF2 activity and oxidative/inflammatory damage in AD.

Supporting this hypothesis, blood biomarker analyses in mild AD patients have revealed differential expressions of numerous inflammatory and redox-related genes, suggesting disruptions in NRF2 and NF-κB functions—both of which are major regulators of redox and inflammation homeostasis [85]. These findings imply that the expression patterns of redox and inflammation-related genes regulated by NRF2 and NF-κB in the blood may serve as valuable biomarkers for diagnosing and monitoring the progression of AD.

In *Nrf2* knockout animals, several functional pathways known to be altered in AD brains are also disrupted [86]. In the APP/PS1 transgenic mouse model, a widely used AD model, NRF2 signaling is impaired during Aβ deposition [87]. Downregulating *Nrf2* in the hippocampus of young senescence-accelerated mice leads to alterations in synaptic plasticity and accelerates cognitive impairments [88]. Overexpression of the TAU P301S mutant protein in a mouse neuroblastoma cell line and primary hippocampal neurons increases KEAP1 levels and enhances KEAP1 K312 acetylation, resulting in reduced NRF2 levels in both the hippocampus and primary neurons [89]. A depletion in NRF2 leads to elevated levels of phosphorylated TAU protein in the hippocampus of mice [62]. The absence of the *Nrf2* gene in AD model mice exacerbates cognitive deficits, including impairments in spatial learning, working memory, and associative memory. The loss of NRF2 function is associated with decreased HO-1 levels, increased BACE1 activity, Aβ production, and elevated interferon-γ (IFN-γ) levels [90,91]. Consistent with these findings, other studies show that the reducing *Nrf2* levels enhances brain inflammation [92].

### 4.2. Pharmacological Modulation of NRF2 in AD

Supporting the role of NRF2 in preventing Aβ-driven AD pathogenesis, genetically engineered compound mutant lines of mice were used [93]. In the study, the AD model *App^NL-G-F/NL-G-F^* knock-in (*App^NLGF^*) mice that harbor three mutations associated with familial AD and exhibit severe accumulation of Aβ and inflammation in the brain were used. NRF2-activated mice generated by hypomorphic *Keap1* gene mutation, referred to as the *KEAP1^FA^* mutation, were also used [94]. Homozygous *KEAP1^FA/FA^* mice and *KEAP1^FA/–^* mice that are heterozygous with *KEAP1^FA^* and *KEAP1* knockout (*KEAP1^–^*) allele were used in the AD model mouse analysis.

When *App^NLGF^* mice are crossed with *KEAP1^FA/FA^* mice, which overexpress NRF2, the compound mutant mice exhibit enhanced glutathione levels, reduced oxidative stress, lower inflammation, and improved cognitive function compared to wildtype mice [75]. Similarly, the administration of 6-(Methylsulfinyl)hexyl isothiocyanate (6-MSITC), an NRF2 inducer, confers these benefits in *App^NLGF^* mice [75]. Enhancing NRF2 signaling via *tert*-butylhydroquinone (tBHQ) treatment or adenoviral *Nrf2* gene transfer also protects AD model mice from Aβ toxicity by upregulating NRF2 target genes and reducing the phosphorylation of p66Shc, a marker of oxidative stress susceptibility [87].

On the pharmacological front, several compounds have been demonstrated to exert anti-AD effects through the activation of NRF2. For instance, dimethyl fumarate (DMF or Techfidera^®^) delays AD progression by enhancing NRF2 signaling, leading to increased expression of antioxidant enzyme and the inhibition of lipid peroxidation, apoptosis, inflammation, mitochondrial dysfunction, and Aβ deposition in embryonic mouse hippocampal neurons [95]. Pre-treatment with DMF preserves cellular viability against Aβ stimulation, reduces TAU hyperphosphorylation, and suppresses NF-κB pro-inflammatory effects in both in vitro and ex vivo systems [96]. In a transgenic mouse model combining amyloidopathy and tauopathy, *Nrf2* deficiency accelerated mortality compared to wild-type mice. *Nrf2* deficiency was associated with increased astrogliosis and microgliosis, indicated by elevating the levels of astrocyte marker *Gfap* and the microglial markers *Iba1* and *Cd11b*. Treatment with DMF decreased inflammatory markers, and improved cognition and motor function in this mouse model [97].

Sulforaphane administration ameliorates cognitive impairment and reduces Aβ accumulation in the brains of 5xFAD mice with five human mutations in APP and PS1 and 3xTg-AD lines with three human mutations in APP, PS1, and TAU [90]. Treatment with sulforaphane improved cognitive function in an Aβ-induced AD acute mouse model in the Y-maze and passive avoidance behavior tests [98]. Additionally, low doses of curcumin analogues efficiently attenuated Aβ-induced oxidative damage in PC12 cells by inhibiting ROS accumulation, activating the KEAP1/NRF2/HO-1 pathway, and modulating apoptosis-related proteins [99].

One of the strongest NRF2 inducers available to date is the synthetic triterpenoid 1-[2-cyano-3,12-dioxooleana-1,9(11-dien-28-oyl)] (CDDO) and its derivatives [100,101]. Among these, omaveloxolone (RTA408), has been approved by the FDA for the treatment of Friedreich’s ataxia. These CDDO derivatives have also been tested for their efficacy in treating AD model mice. In the Tg19959 AD model mice, which harbors the human amyloid precursor protein with two mutations, administration of CDDO-methylamide (CDDO-MA) notably enhances spatial memory retention and decreases plaque accumulation, Aβ42 levels, microgliosis, and oxidative stress [102]. The treatment of APP/PS1 mice with RTA408 reduces Aβ plaque load, rescues neurons, increases antioxidant capacity, and enhances cognitive function [103]. In addition, CDDO-4(-pyridin-2-yl)-imidazole (CDDO-2P-Im) has been shown to effectively cross the blood–brain barrier and reach the mouse brain. CDDO-2P-Im treated APP/TAU mice showed a decrease in Aβ42 levels, but not Aβ40 levels [104]. Taken together, these findings support that NRF2 plays a significant role in protecting against the pathogenesis of AD (Table 1). Studies in mouse models lead us to propose that activating the NRF2 signaling pathway may help alleviate AD pathology.

Most of NRF2 activators have demonstrated the ability to cross the BBB [104,105,106,107] and improve brain functions, thereby confirming their capacity to reach the brain [75,108]. However, the molecular off-target effects and associated unpredictable side-effects present significant obstacles for clinical trials and regulatory approval. Most common NRF2 activators work by inhibiting KEAP1 to prevent NRF2 proteasomal degradation. While KEAP1 is a promising target for addressing NRF2 defects, therapeutic targeting is challenging due to KEAP1′s complex interactions with numerous proteins involved in processes like DNA replication, cytoskeletal dynamics, transcription, and apoptosis, raising concerns about potential side effects [109].

For instance, the phase III BEACON trial on bardoxolone methyl (CDDO-Me), conducted in patients with type 2 diabetes mellitus and stage 4 chronic kidney disease, revealed that while the drug did not reduce the risk of end-stage renal disease, it also significantly increased the incidence of heart failure events [110]. To mitigate the cardiovascular risks with CDDO-Me, Cathepsin B-cleavable prodrugs have been developed to enhance water solubility and achieve selective activation, reducing off-target effects [111]. Additionally, researchers are now exploring sustained-release systems like nanoparticles to maintain drug levels over time. For example, antioxidant response activating nanoparticles (ARAPas) deliver CDDO-Me to atherosclerotic plaques, preventing degradation and enabling controlled release for improved efficacy [112]. However, the concentration of NRF2 activators is a critical factor, as it may influence their potential to modify multiple residues within Keap1 and other proteins [113,114], thereby increasing the probability of unintended effects.

Furthermore, small molecule protein–protein interaction (PPI) inhibitors have been developed to overcome the unpredictable off-target effects [115]. These non-covalent compounds are highly specific and bind reversibly, potentially offering a better safety profile [116]. Nevertheless, the development of KEAP1-NRF2 PPI inhibitors as therapeutic agents for AD faces significant difficulty, including poor BBB penetration, high polarity, low stability, and limited bioavailability [117,118].

## 5. Roles of NRF2 in Astrocyte, Neuron and Vasculature in AD

### 5.1. Impact of NRF2 in Astrocytes on AD

Given the antioxidative and anti-inflammatory functions of NRF2, therapeutic strategies targeting NRF2 could potentially inhibit microglia-mediated neuroinflammation. Recent studies have also highlighted the importance of NRF2 in astrocytes in the context of AD. Activating NRF2 in astrocytes has emerged as a promising therapeutic strategy for addressing both acute neuronal injury and chronic neurodegenerative conditions driven by oxidative stress [119].

Studies in co-culture systems of astrocytes and neurons have shown that GSH depletion in astrocytes eliminates their neuroprotective effects [120,121]. Astrocytes that raise GSH synthesis efficiency compared to neurons maintain elevated GSH levels because of the NRF2-driven activation of GSH-synthesizing enzymes such as GCLC and GCLM [122]. This NRF2-mediated regulation ensures a steady release of GSH, which is crucial for protecting neighboring neurons from oxidative stress [123,124,125].

In the 3xTg-AD mouse model, a significant reduction in the GSH ratio was observed in brain tissue homogenates [126]. Under inflammatory stimulation, control astrocytes significantly increased GSH secretion, whereas AD astrocytes exhibited an impaired response and failed to enhance GSH secretion. However, NRF2 activation restored the secretory capacity of AD astrocytes, leading to enhanced GSH secretion, slight increases in cellular GSH levels, and reductions in amyloid secretion and cytokine release [127]. These results highlight the critical role of NRF2 in astrocytes for combating oxidative stress and neuroinflammation in AD.

Astrocytes regulate inflammatory responses, reduce Aβ burden, and maintain BBB integrity, all of which contribute to a neuroprotective environment [128,129]. Pharmacological or genetic modulation of astrocyte activity has alleviated neuroinflammation and improved cognitive function in AD mouse models [128,129]. However, the dual role of astrocytes in AD progression remains complex; reactive astrocytes can either mitigate inflammation or exacerbate neurotoxicity under certain conditions [130,131]. Astrocytes from AD patients exhibit altered cytokine secretion (e.g., IL-2, IL-6, and IL-10) in response to inflammation and rely more on oxidative metabolism, leading to increased ROS production compared to healthy astrocytes [132].

In the context of astrocytes, NF-κB serves as a pivotal regulator of pro-inflammatory and oxidative pathways, directly influencing neuroinflammation and neurotoxicity [133]. The absence of astrocytic NF-κB leads to reduced chemokine levels and oxidative stress in the injured nervous system [134,135]. The interaction between NRF2 and NF-κB is particularly important in regulating immune responses and disease progression. Studies on astrocytes from *Nrf2* wild-type and knockout mice have shown that the absence of *Nrf2* amplifies NF-κB activity, increasing inflammatory cytokines (TNF-α, IL-1β, IL-6, and MMP9) and NF-κB DNA-binding activity following injury [136]. This highlights NRF2′s critical role in counteracting NF-κB-driven inflammation.

AD models further support the neuroprotective role of NRF2. The 3xTg-AD mouse model and AD patient brains exhibit reduced *Nrf2* levels in hippocampal astrocytes [81,137]. In the 5xFAD AD model, the absence of *Nrf2* leads to reactive astrocyte activation, while enhancing *Nrf2* expression impedes NF-κB p65 recruitment to neurotoxic astrocyte genes, reducing their expression. ChIP-seq analysis shows that NRF2 activation shifts astrocyte function toward anti-inflammatory and neuroprotective pathways by increasing its binding while reducing NF-κB binding at inflammatory gene loci [138]. Treatment with NRF2 inducers, such as sulforaphane, has been shown to reduce neurotoxic astrocytes and improve cognitive behavior in AD mice [138].

Astrocytes express high levels of glycogen and glycolytic enzymes, showing significant glycolytic activity to meet the brain’s energy demands [139]. During astrocytic glycolysis, glucose is rapidly converted to lactate and transferred to neurons, providing a quick and efficient energy source for neurons, particularly for energy-demanding functions like long-term plasticity and memory formation [131,140]. NRF2 induction in induced pluripotent stem cell (iPSC)-derived astrocytes further enhances this glycolytic activity, as indicated by increased extracellular acidification rate, promoting neuronal energy supply under both normal and AD conditions [127].

A research group conducted a detailed analysis of gene alteration in astrocytes within APP/PS1 β-amyloidopathy and MAPT^P301S^ tauopathy mouse models [141]. The *Nrf2* gene was found to be significantly enriched in astrocytes in both Aβ and TAU pathology models. To further investigate the role of NRF2, the researchers crossed the Aβ/TAU pathology mice with mice engineered to overexpress NRF2 specifically in astrocytes. The results demonstrated that astrocytic NRF2 overexpression led to a reduction in phosphorylated TAU accumulation and mitigated neurodegeneration in the brain of tauopathy mice. Additionally, astrocytic NRF2 reduced both the number and area of Aβ plaque in Aβ pathology mice. In both AD mouse models, NRF2 overexpression not only alleviated transcriptional disruptions but also rescued cognitive deficits, underscoring its therapeutic potential [141]. To conclude, the influence of astrocytic NRF2 may help prevent AD progression and preserve brain function. However, future studies are needed to determine whether activating NRF2 in astrocytes at later stages of the disease could yield similar protective effects.

### 5.2. Impact of NRF2 in Neurons in AD

Despite the neuron exhibiting the lowest *NRF2* mRNA expression compared to microglia and astrocyte in the brain, several studies indicate that neuronal NRF2 may be involved in AD. Furthermore, activation of NRF2 has been shown to protect neural cells from Aβ-peptide-induced neurotoxicity [87,142] and TAU pathology in vitro [143]. Induction of NRF2 activation with tBHQ protected isolated neurons from Aβ-induced cell death in AD mice [87]. AD neurons also exhibit reduced mitochondrial function and number [144], with evidence suggesting that alterations in mitochondrial energy production precede cognitive decline [145]. NRF2 activation by *Centella asiatica* was shown to enhance mitochondrial function and reduce oxidative stress in hippocampal neurons exposed to Aβ [146]. Treatment with sulforaphane in immortalized cortical neurons prevented mitochondrial dysfunction induced by caspase-3 cleaved TAU and reduced ROS levels, indicating the potential of NRF2 activation in preserving neuronal function under TAU-related challenges [143].

However, NRF2 in hippocampal neurons in AD is predominantly cytoplasmic, suggesting reduced nuclear translocation or nuclear presence. Downregulation of ARE genes was also observed in AD neurons [81], and nuclear extracts from frontal cortex cells confirmed decreased NRF2 levels in AD patients, supporting the idea that NRF2 may be ineffective in responding to oxidative stress in AD neurons [81].

### 5.3. Neuro-Endothelial Cell Contribution of NRF2 in AD

NRF2 is well-recognized for its protective role in cerebrovascular health [50,120,147,148]. Endothelial cells (ECs) play a key role in regulating vasomotor tone by releasing vasoactive agents, such as nitric oxide (NO) and adenosine. NRF2 activation has been shown to restore EC function, supporting vascular health and neurovascular coupling [122]. For instance, overexpression of NRF2 in human aortic ECs suppressed TNF-α-induced cytotoxicity while enhancing the expression of antioxidant proteins such as HO-1 and glutathione [149]. Similarly, sulforaphane pretreatment in mouse brain microvascular ECs induced NRF2-driven enzymes, including HO-1 and GCLM, which mitigated oxidative stress and limited free radical generation during reoxygenation [150]. These studies demonstrate that NRF2 activation is vital for protecting brain microvascular ECs against oxidative damage.

NRF2 also plays a crucial role in maintaining the integrity of the BBB, as evidenced by multiple experimental models. In vitro studies show that *NRF2* knockout leads to BBB disruption, characterized by reduced expression of tight junction proteins such as ZO-1, occludin, and claudin-5, along with the adherens junction protein VE-cadherin [151,152]. This disruption underscores the importance of NRF2 in preserving BBB structural integrity. Promoter analyses further indicate that NRF2 can directly regulate the claudin-5 gene, reinforcing BBB integrity [152]. In VCID (vascular contributions to cognitive impairment and decline) mouse models, sulforaphane treatment enhanced the expression of tight junction proteins, claudin-5 and occludin, demonstrating a protective effect on the BBB [152]. These findings suggest that NRF2 activation can mitigate vascular dysfunction in AD by reducing oxidative stress and maintaining BBB integrity.

## 6. Regulation by NRF2 of Microglia Activity in AD

### 6.1. Role of Microglia in CNS Homeostasis and Immune Response

Microglia are the innate immune cells of the CNS, playing a critical role in maintaining CNS homeostasis. Microglia develop from progenitor cells in the embryonic yolk sac, migrate to the brain rudiment, and persist into adulthood [153,154]. Transforming growth factor-β (TGF-β) and colony stimulating factor 1 receptor (CSF1R) signaling regulate the homeostasis and self-renewal of microglia [155,156]. Microglia contribute to CNS integrity by responding to injury, defending against pathogens, and shaping neural circuits during development through the removal of unnecessary neurons and synapses [157,158,159,160].

In the brain, the development and resolution of inflammation are primarily governed by microglia, which constitute up to 20% of the brain’s cellular population. While microglial activation is crucial for the brain’s defense during neuroinflammation, excessive activation can lead to or exacerbate neuronal damage, particularly in neurodegenerative diseases and aging. This dual role highlights their importance in both physiological and pathological conditions [161].

The traditionally binary classification of microglia as “M1” (pro-inflammatory and neurotoxic) or “M2” (anti-inflammatory and neuroprotective) [162,163] is now regarded as overly simplistic. Recent research demonstrates that microglia exist in a spectrum of states dynamically influenced by their microenvironment [164]. These states are regulated by complex transcriptional, epigenetic, and metabolic regulations, allowing microglia to adapt to varying physiological and pathological contexts. We summarize the various terminology and characteristics of microglia in Table 2.

Microglia exhibit reactive states in response to challenges such as Aβ or α-synuclein deposition, which are common features of neurodegenerative diseases [165,166]. One extensively studied microglial state is the disease-associated microglia (DAM) phenotype, which was first identified in the 5xFAD mouse model [167] and has since been observed in human AD [168] and multiple sclerosis (MS) [169,170]. DAMs undergo a two-step activation process: an initial TREM2-independent phase involving checkpoint downregulation, followed by TREM2-dependent activation [167]. DAMs cluster near Aβ plaques, participating in their clearance and exhibit a distinct transcriptional profile, including the upregulation of genes such as *APOE*, *AXL*, *SPP1*, and *TREM2*, alongside the downregulation of *CX3CR1* and *P2RY12* [168].

Another important state is the microglial neurodegenerative (MGnD) phenotype, seen in models of amyotrophic lateral sclerosis (ALS), MS, and AD. MGnD microglia, also associated with the TREM2-APOE pathway, display disrupted homeostasis. Targeting this pathway has been shown to restore microglial functions and prevent neuronal loss in models of acute neurodegeneration [171].

The dark microglial (DM) state is characterized by distinct morphological changes, such as mitochondrial damage, an expanded Golgi apparatus, and cell shrinkage. DMs are associated with non-homeostatic conditions such as aging, chronic stress, fractalkine signaling deficiency, and AD pathology [172,173]. DMs express high levels of CD11b and TREM2 and are often found near synaptic clefts in regions of amyloid deposition [174].

Lipid droplet-accumulating microglia (LDAMs) are particularly prevalent in the aged hippocampus, where they constitute more than 50% of the microglial population [175,176]. LDAMs display defective phagocytic activity and secrete pro-inflammatory cytokines, contributing to neuroinflammation. These microglia produce high levels of ROS and show a transcriptional profile distinct from DAMs and MGnD, with no upregulation of genes, such as *TREM2* and *APOE* [177]. This suggests that LDAMs represent a dysfunctional microglial state associated with aging and related impairments.

Proliferative-region-associated microglia (PAMs) are predominantly found in the developing CNS, particularly in regions undergoing myelination, such as the corpus callosum and cerebellar white matter. PAMs display high metabolic activity, an amoeboid morphology, and actively engulf newly formed oligodendrocytes [178,179]. Unlike DAMs, PAMs rely on unique cytokines and trophic factors rather than the TREM2-APOE pathway, suggesting their involvement in early development and impaired myelination conditions.

These wide-range findings suggest that microglia exhibit plasticity in response to different pathologies, highlighting the importance of identifying disease-specific microglial states (Table 2). Understanding the factors that influence these states will be critical for developing effective treatments for AD and other neurodegenerative disorders.

### 6.2. Regulatory Influence of NRF2 in Microglia

It has been reported that the activation of NRF2 target genes in microglia reduces oxidative stress levels and thereby regulates the neuroinflammatory response [78,180,181,182]. The roles of NRF2 in modulating the innate immune system have been demonstrated in models of acute inflammation induced by LPS [180,183,184,185]. Under LPS treatment, sulforaphane, an NRF2 inducer [186], significantly reduces the expressions of interleukins IL-1β, IL-6, and inducible nitric oxide synthase (iNOS) in BV2 microglia cells and primary mouse microglia. Furthermore, sulforaphane has been shown to suppress microglial activation by increasing HO-1 expression and decreasing proinflammatory cytokines IL-6 and TNF-α in the hippocampus [180,181,182]. Intriguingly, conditioned medium from activated microglia upregulates the NRF2-mediated antioxidant defenses in cultured astrocytes [187], suggesting the presence of paracrine regulation between these two glial cell types. Indeed, factors released by activated microglia promote histone deacetylation in astrocytes; however, histone deacetylase inhibitors or GSK3β inhibitor can restore NRF2 signaling and counteract the inflammation-induced suppression of astrocyte activity [187,188].

It has been reported that NRF2 activation enhances antioxidant capacity and suppresses inflammatory responses by inhibiting NF-κB activation in BV2 microglia cells [189,190]. Treatment of LPS-stimulated BV2 cells with the NRF2 inducer RTA408 [191] inhibited NF-κB/IL-6 signaling and reduced neuroinflammation [103]. In *Nrf2*-deficient mouse primary microglia, higher levels of p65 nuclear translocation were observed even in the resting state. The NRF2 inducer DMF [192] was shown to suppress inflammatory activation, microglia-mediated neurotoxicity and NF-κB activation in mouse primary microglia [193]. These findings provide strong evidence for crosstalk between the NRF2 and NF-κB signaling pathways in microglia.

TREM2 is a cell surface receptor that belongs to the lectin-like immunoglobulin superfamily. TREM2 is expressed at higher levels in microglia than in neurons and plays a critical role in regulating the inflammatory response [194]. It has been found that NRF2 binds to the regulatory region of the *Trem2* gene and regulates its transcription in BV2 cells. Activation of NRF2 enhances *Trem2* expression, which leads to attenuation of depressive behaviors in the chronic social defeated stress mouse model [195]. In an intracerebral hemorrhage mouse model and in BV2 cells, NRF2 induction upregulated the expression of *Trem2*, *Cd206* and *Bdnf* genes, while reducing the expression of *Trem1*, *Tnf* and *Cd80* genes. This indicates that NRF2 promotes microglial phenotype transformation toward an anti-inflammation and phagocytosis state, contributing to hematoma clearance and neurological recovery after intracerebral hemorrhage [196]. Taken together, these findings provide strong evidence that the NRF2 system is essential for regulating neuroinflammation in response to oxidative stress in the brain.

### 6.3. NRF2 Regulation of Microglial States in AD

**Figure 4:**
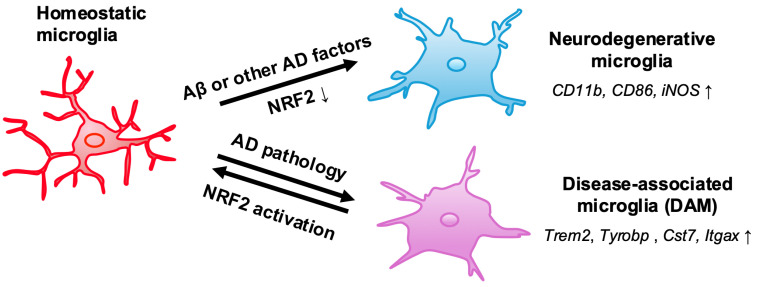
NRF2 regulation of microglial states in AD. The downregulation of NRF2 in AD microglia enhances the expression of neurodegenerative phenotype markers, such as *CD11b*, *CD86*, and *iNOS*. This phenomenon has been observed in AD mini-brains, AD mouse models, and AD patients [190]. Conversely, the activation of NRF2 in AD mice leads to a reduction in the expression of disease-associated microglia (DAM) markers, including *TREM2*, *TYROBP*, *CST7*, and *ITGAX* [76]. These findings collectively underscore the crucial role of NRF2 in regulating the phenotypic transition of microglia within the context of AD, suggesting that enhancing NRF2 signaling could be a potential therapeutic strategy to modulate neuroinflammation and improve outcomes in AD.

The relevance of NRF2 in modulating the innate immune system has been discussed, and an increasing number of studies report the participation of NRF2 in modulating microglial phenotypes in AD (Figure 4).

In both the cortices and the hippocampus of *App^NLGF^::Keap1^FA/FA^* and *App^NLGF^::Keap1^FA/–^* mouse brains, NRF2 suppresses the transition from homeostatic microglia to DAMs [75,197]. While the expression levels of the homeostatic microglial markers, e.g., *Cx3cr1*, *P2ry12*, are slightly elevated in the cortex and hippocampus in *App^NLGF^* mice, these expressions cannot be reduced by NRF2 activation. In contrast, NRF2 activation suppresses the expression of both stage 1 DAM markers, such as *Trem2* and *Tyrobp*, and stage 2 DAM markers, such as *Cst7* and *Itgax*, in *App^NLGF^* mice. *App^NLGF^::Keap1^FA/FA^* mice also show lower proinflammatory responses and fewer phagocytic cells compared to *App^NLGF^* mice. These observations imply that microglial activation can be suppressed by NRF2 induction. Supporting this notion, a series of behavior analyses showed that genetic NRF2 induction improves impaired cognition. These findings suggest that NRF2 is involved in the shift between homeostatic microglia and DAMs, highlighting the potential of NRF2 inducers as therapeutic targets for AD pathology [75].

Another study further demonstrates the critical role of NRF2 in microglial phenotype transition by monitoring microglial markers in reconstituting AD models, specifically using 3D human-AD mini-brains on a microfluidic platform. This platform co-cultures human neuroprogenitor-derived neurons with APP mutations (hNPCs), astrocytes, and human iPSC-driven microglia [198]. A key finding in this experiment is that IFN-γ-mediated downregulation of NRF2 promotes ROS accumulation and drives the transition of neuroprotective microglia to a neurodegenerative phenotype via the classical NF-κB pathway, leading to neurodegeneration in the AD mini-brains. In contrast, restoring NRF2 levels in microglia prevents their activation into a neurodegenerative state and significantly inhibits tauopathy. These results are further corroborated by analyses of brain samples from AD patients and 5xFAD AD mice [199]. These findings suggest that the IFN-γ-driven downregulation of NRF2 in microglia is a crucial target to ameliorate AD pathology.

### 6.4. NRF2 and Ferroptosis in AD Microglia

Microglia are key players in sequestering excess iron in response to acute insults [200,201]. Ferroptosis, a newly recognized form of iron-dependent programmed cell death, is distinct from other cell death mechanisms. Recent studies have shown that ferroptosis is integral to the pathology of AD. Iron dyshomeostasis contributes to the deposition of Aβ plaques and the formation of neurofibrillary tangles [202]. Consequently, iron accumulation becomes a hallmark of AD [203]. Recent research showed that iron loading in iPSC-derived microglia causes microglial activation, altering phagocytosis ability and a strong upregulation of NRF2 signaling [204].

**Figure 5:**
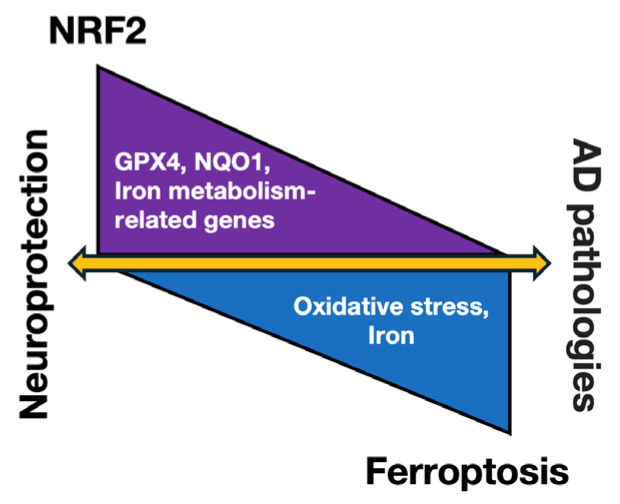
Schematic representation of NRF2-mediated neuroprotection and ferroptosis in AD. The relationship between ferroptosis and AD pathologies is increasingly being recognized. Activation of the NRF2 pathway is associated with neuroprotection in the brain, primarily through the upregulation of antioxidant genes such as GPX4 and NQO1, as well as genes involved in iron metabolism. Oxidative stress and iron deposition in the brain can promote ferroptosis, which exacerbates AD pathologies. The correlation between NRF2 activation and reduced ferroptosis activity suggests that NRF2 may serve as a potential therapeutic target for mitigating neurodegeneration in AD, in part by preventing ferroptosis.

The levels of several NRF2-regulated proteins, such as GPX4-an enzyme involved in GSH biosynthesis, are closely linked to ferroptosis. GPX4, NQO1, and various iron metabolism-related proteins have been found to be altered in AD [205,206]. Activated NRF2 combats oxidative stress induced by excess iron, thereby protecting tissues and cells from ferroptosis [207]. Furthermore, reduced levels of *NRF2* mRNA in the brains of AD patients is involved in ferroptosis in AD pathology (Figure 5). Thus, by regulating the genes related to iron and the glutathione metabolism, NRF2 significantly impacts the development of AD [202].

### 6.5. NRF2 Regulates NVU Functions Through Microglia in AD

Microglia are integral components of the NVU, and regulate vascular tone, cerebral blood flow, and vasodilatory responses in capillaries, highlighting their importance in maintaining cerebrovascular health [208]. Emerging evidence links microglia and the vascular system to AD risk. Many AD-associated risk genes expressed in the brain vasculature are microglia-specific in mice, suggesting an evolutionary relationship between microglial function and vascular health in AD pathology [29]. In AD mouse models, the elimination of microglia leads to increased vascular hemorrhage and Aβ deposition, underscoring their protective role in maintaining vascular stability [209]. Systemic inflammation has also been shown to drive microglial clustering around blood vessels [210], which influences BBB integrity and function [211]. In a VCID mouse model, the absence of NRF2 exacerbated white matter damage, impaired vascular function, and accelerated microgliosis. These findings demonstrate that NRF2 not only supports vascular integrity but also modulates microglial activity in neurovascular contexts [212].

## 7. Conclusions

**Figure 6:**
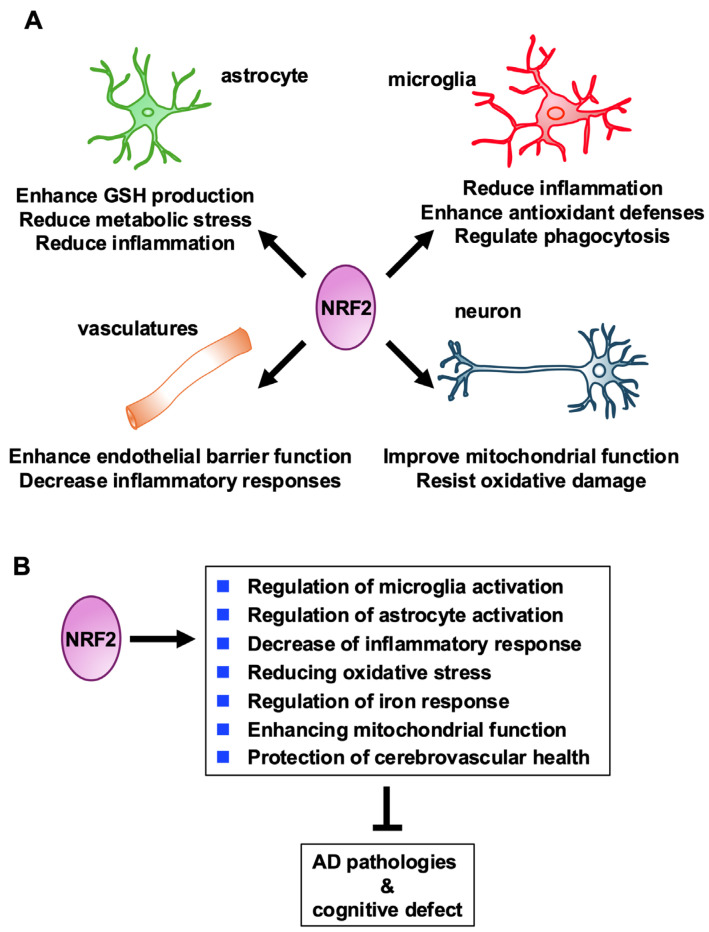
Schematic representation of NRF2 functions in CNS. (**A**) Cell type-specific functions of NRF2 in CNS. Diverse roles of NRF2 in three distinct cell types within the CNS and vasculature are shown. Note that in microglia, NRF2 reduces inflammation, enhances antioxidant defenses, and regulates phagocytosis, contributing to immune homeostasis and neuroprotection. In astrocytes, NRF2 promotes glutathione (GSH) production, alleviates metabolic stress, and reduces inflammation, supporting neuronal survival and CNS health. In neurons, NRF2 improves mitochondrial function and resistance to oxidative damage, protecting against neurodegeneration. In the vasculature, NRF2 enhances endothelial barrier function and mitigates inflammatory responses, maintaining vascular integrity and reducing neurovascular dysfunction. Collectively, NRF2 serves as a central regulator of oxidative stress and inflammation across multiple CNS cell types and the vascular system, highlighting its therapeutic potential in neurodegenerative diseases such as AD. (**B**) Schematic representation of NRF2 regulation in the CNS. When NRF2 activated in the CNS, NRF2 regulates the activation of microglia and astrocytes, leading to a decrease in inflammatory responses and a reduction in oxidative stress. Additionally, NRF2 plays a vital role in maintaining iron homeostasis, enhancing mitochondrial function and protection of cerebrovascular health. Together, these effects of NRF2 activation underscore its significant influence in mitigating AD pathology by modulating cellular responses and processes.

As summarized in Figure 6A, we have described how NRF2 plays crucial roles in each cell type in CNS under the homeostatic and AD conditions. NRF2 expression levels are higher in microglia compared to astrocytes and neurons in both mice and humans. Therefore, certain inconsistencies in the results of targeting the NRF2 pathway reported in the literature might be attributed to the specific cell types involved. NRF2 activation in microglia appears to be a promising therapeutic target for AD.

As shown in Figure 6B, the roles of NRF2 in regulating antioxidant and anti-inflammatory responses make this transcription factor a key player in mitigating the neuroinflammation and oxidative stress associated with AD—two hallmarks of AD pathogenesis. Microglia are hyperactivated in AD, contributing to chronic inflammation and oxidative damage that exacerbate neuronal loss. The activation of NRF2 signaling in microglia enhances the expression of antioxidant enzymes, reduces pro-inflammatory factors, promotes cell survival, and improves mitochondrial function. These NRF2-mediated actions help in alleviating the pathological progression of AD. While NRF2 activation in astrocytes, neurons, and vasculature also contributes to reducing metabolic stress, oxidative damage, and vascular dysfunction, the microglia-specific benefits of NRF2 remain central to its therapeutic potential in AD.

In addition, various compounds that activate NRF2 have been identified as the potential therapeutic agents for AD. For instance, DMF, CDDO derivatives and other types of NRF2 inducers have been shown to attenuate AD pathology in both in vitro and in vivo studies. Targeting NRF2 in microglia offers a promising strategy for developing therapeutic interventions aimed at reducing oxidative stress and inflammation in AD, potentially slowing or halting disease progression.

## Figures and Tables

**Table 1:** Summary of NRF2 activators and their therapeutic effects in AD models.

NRF2 Activator	Mechanism	AD Models Used	Therapeutic Effects Observed
CDDO derivatives (e.g., Omaveloxolone, CDDO-MA)	Covalently modify KEAP1 cysteine residues; enhance NRF2 stability and antioxidant defense in the CNS	Tg19959, APP/PS1 mouse models	Improved spatial memory, reduced Aβ plaques and Aβ42 levels, enhanced antioxidant defenses, and decreased oxidative stress
Sulforaphane	Activates NRF2 by modifying cysteine residues on KEAP1, preventing NRF2 degradation	5xFAD, 3xTg-AD mouse models	Reduced cognitive impairment, Aβ accumulation, and ROS generation; improved BBB integrity
Dimethyl fumarate (DMF)	Alters KEAP1 through electrophilic modification, leading to NRF2 stabilization and activation	Embryonic mouse hippocampal neurons, APP/TAU mice	Suppressed tau phosphorylation, reduced NF-κB activation, and improved mitochondrial function
6-MSITC	Enhances NRF2 signaling and glutathione production	*App^NLGF^* knock-in mice	Lowered oxidative stress and inflammation; improved cognitive function
tBHQ	Inhibits KEAP1-mediated NRF2 degradation, allowing nuclear translocation of NRF2	APP/PS1 mouse models	Protection against Aβ toxicity, reduced phosphorylation of stress markers, and improved cognition
Curcumin analogues	Induces KEAP1/NRF2/HO-1 pathway activation, inhibits ROS, and regulates apoptosis-related proteins	PC12 cell culture models	Attenuated oxidative damage and apoptosis

This table highlights key NRF2 activators in special reference in their mechanisms of action, the AD models used, and the therapeutic effects observed. It provides an overview of how various compounds enhance NRF2 activity, reduce oxidative stress, and mitigate AD pathology, emphasizing their potential as therapeutic agents.

**Table 2:** Phenotypes of microglia.

Phenotypes	Key Features	Relevance to AD
M1 Microglia	Pro-inflammatory and neurotoxic Markers: CD11b, CD86, iNOS	Associated with increased neuroinflammation in AD
M2 Microglia	Anti-inflammatory and neuroprotective Markers: ARG1 and CD206	Support tissue repair and neuroprotectionReduced in AD microenvironments
Disease-Associated Microglia (DAMs)	Observed near Aβ plaques Markers: APOE, AXL, TREM2	Play a role in plaque clearance but transitions to dysfunction under chronic stress
Microglial Neurodegenerative Phenotype (MGnD)	Linked to the TREM2-APOE axis Shows disrupted homeostasis	Associated with neuronal loss in AD
Dark Microglia (DMs)	Characterized by mitochondrial damage, expanded Golgi, and synaptic cleft localization Highly active	Found in aging and chronic stressProminent in AD pathology
Lipid-Droplet Accumulating Microglia (LDAMs)	Found predominantly in the aged hippocampus Impaired phagocytosis ROS production	Linked to heightened neuroinflammation and aging-related neurodegenerative processes
Proliferative-Region Associated Microglia (PAMs)	Observed in myelination areas Amoeboid morphology High metabolic activity	Present in developmental stages and conditions with impaired myelination

Distinct phenotypes of microglia based on their key features and relevance to AD.
